# Arterial Embolization Hyperthermia Using As_2_O_3_ Nanoparticles in VX_2_ Carcinoma–Induced Liver Tumors

**DOI:** 10.1371/journal.pone.0017926

**Published:** 2011-03-23

**Authors:** Hui Yu, Guang-Yu Zhu, Rui-Zhi Xu, Huan-Zhang Niu, Qin Lu, Guo-Zhao Li, Zi-Yu Wang, Dong-Sheng Zhang, Ning Gu, Gao-Jun Teng

**Affiliations:** 1 Jiangsu Key Laboratory of Molecular Imaging and Functional Imaging, Department of Radiology, Zhong-Da Hospital, Medical School of Southeast University, Nanjing, China; 2 Jiangsu Laboratory for Biomaterials and Devices, State Key Laboratory of BioElectronics, School of Biological Science and Medical Engineering, Southeast University, Nanjing, China; 3 Department of Pathology and Pathophysiology, Medical School of Southeast University, Nanjing, China; National Institutes of Health, United States of America

## Abstract

**Background:**

Combination therapy for arterial embolization hyperthermia (AEH) with arsenic trioxide (As_2_O_3_) nanoparticles (ATONs) is a novel treatment for solid malignancies. This study was performed to evaluate the feasibility and therapeutic effect of AEH with As_2_O_3_ nanoparticles in a rabbit liver cancer model. The protocol was approved by our institutional animal use committee.

**Methodology/Principal Findings:**

In total, 60 VX_2_ liver-tumor-bearing rabbits were randomly assigned to five groups (*n* = 12/group) and received AEH with ATONs (Group 1), hepatic arterial embolization with ATONs (Group 2), lipiodol (Group 3), or saline (Group 4), on day 14 after tumor implantation. Twelve rabbits that received AEH with ATONs were prepared for temperature measurements, and were defined as Group 5. Computed tomography was used to measure the tumors' longest dimension, and evaluation was performed according to the Response Evaluation Criteria in Solid Tumors. Hepatic toxicity, tumor necrosis rate, vascular endothelial growth factor level, and microvessel density were determined. Survival rates were measured using the Kaplan-Meier method. The therapeutic temperature (42.5°C) was obtained in Group 5. Hepatotoxicity reactions occurred but were transient in all groups. Tumor growth was delayed and survival was prolonged in Group 1 (treated with AEH and ATONs). Plasma and tumor vascular endothelial growth factor and microvessel density were significantly inhibited in Group 1, while tumor necrosis rates were markedly enhanced compared with those in the control groups.

**Conclusions:**

ATON-based AEH is a safe and effective treatment that can be targeted at liver tumors using the dual effects of hyperthermia and chemotherapy. This therapy can delay tumor growth and noticeably inhibit tumor angiogenesis.

## Introduction

Hepatocellular carcinoma (HCC) is one of the most common fatal malignant tumors worldwide [1]. Surgical removal remains the gold standard for liver cancer. However, the majority of tumors are inoperable because diagnosis is seldom established in the early stages [2], [3]. Hyperthermia, a therapeutic procedure that raises the temperature of a tumor, has attracted increasing attention as a promising approach to cancer therapy [4]–[6]. The rationale for the use of hyperthermia is that sustained temperatures above 42.5°C directly kill living cells. Compared with chemotherapy and radiotherapy, hyperthermia has fewer side effects.

Transcatheter arterial chemoembolization (TACE) has been established as a nonsurgical treatment for HCC, and many studies have established its safety and efficacy, with long-term survival rates comparable to those seen following surgical resection [2], [7]. The rationale for this procedure is that the normal liver parenchyma receives two-thirds of its blood supply from the portal vein system, whereas tumors in the liver derive virtually all of their blood supply from the hepatic arterial system. Thus, for hepatic tumors, substances injected into the hepatic arterial system will be preferentially delivered to the liver tumor. This same principle can be used in arterial embolization hyperthermia (AEH), which has been used as a localized modality of hyperthermia for many years and has shown promise in recent experimental animal studies. In AEH, tumor tissue is heated specifically, sparing surrounding normal hepatic tissue [8]–[10].

Arsenic trioxide (As_2_O_3_) has been successfully used to treat patients with acute promyelocytic leukemia [11]. Recent studies have shown that As_2_O_3_ has antitumor activity towards many solid tumors, including hepatomas [12]–[14]. Its mechanisms of action include induction of apoptosis of various cancer cell lines [15], inhibition of tumor growth [16], and angiogenesis [17].

The purpose of the present study was to evaluate the safety and the anticancer effects of the use of As_2_O_3_ nanoparticles combined with AEH in established transplanted rabbit VX_2_ hepatic tumors.

## Results

### Technical Success

The TAE and CT procedures were successfully performed in all animals with the exception of two rabbits. One rabbit in Group 1 died during general anesthetic induction within the scheduled CT follow-up on day 3 after TAE, and another rabbit in Group 4 died 6 h after a sham procedure on Day 1. Both animals were excluded from analyses.

### Morphology of ATONs

**Figure 1:**
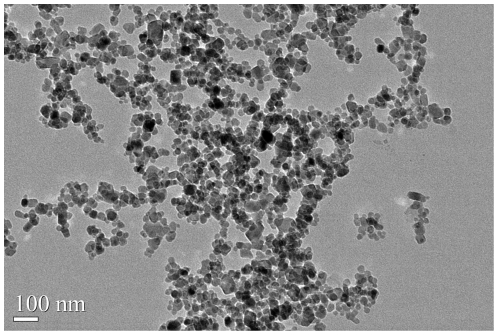
TEM micrographs of ATONs. Transmission electron microscopy showed ATONs to be approximately spherical, with diameters of about 40 nm.

**Figure 2:**
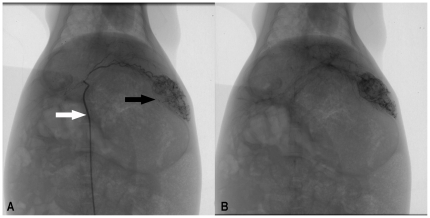
Representative rabbit hepatic angiographic images. Figure 2A Selective placement of 3F microcatheter (white arrow) and injection of ATONs suspended in lipiodol in the proper left hepatic artery revealed ill-defined hypervascularity tumor staining in the left lobe. Figure 2B Postembolization images show complete stasis of blood flow and demonstrate dense staining of the tumor bed, suggesting successful delivery and excellent distribution of the magnetic nanoparticle fluid mixture in the tumor.

**Figure 3:**
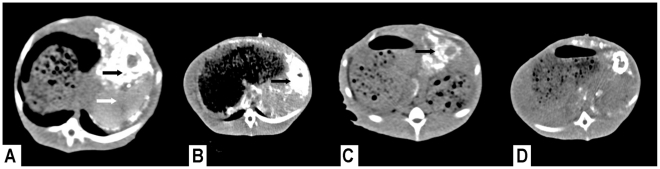
Serial CT imaging findings after TAE. Figure 3A CT scan obtained immediately after TAE. ATONs have accumulated primarily in the tumor (black arrow), whereas ATON retention in the NHP region (white arrow) is minimal. Figure 3B CT scan obtained 3 days after TAE shows ATONs retained in the tumor (black arrow). The number of ATONs within the NHP region is decreasing. Figure 3C Seven days after TAE (4 days after AEH), ATONs are selectively retained in the tumor (black arrow), whereas they are barely visible within the NHP region. Figure 3D Fourteen days after TAE (11 days after AEH), ATONs are still observed in the tumor region, and have especially accumulated in the peripheral area.

Transmission electron microscopy (Fig. 1) showed ATONs to be approximately spherical, with diameters of about 40 nm. Immediately after intra-arterial injection (Fig. 2A, B), lipiodol or ATONs suspended in lipiodol were concentrated in the margin of the tumor, mainly in vascular channels. Even 2 weeks after intra-arterial injection, retention of lipiodol or ATONs persisted, suggesting that clearance of the agent was much faster from normal liver than from tumors (Fig. 3A–D).

### Heating Curve

**Figure 4:**
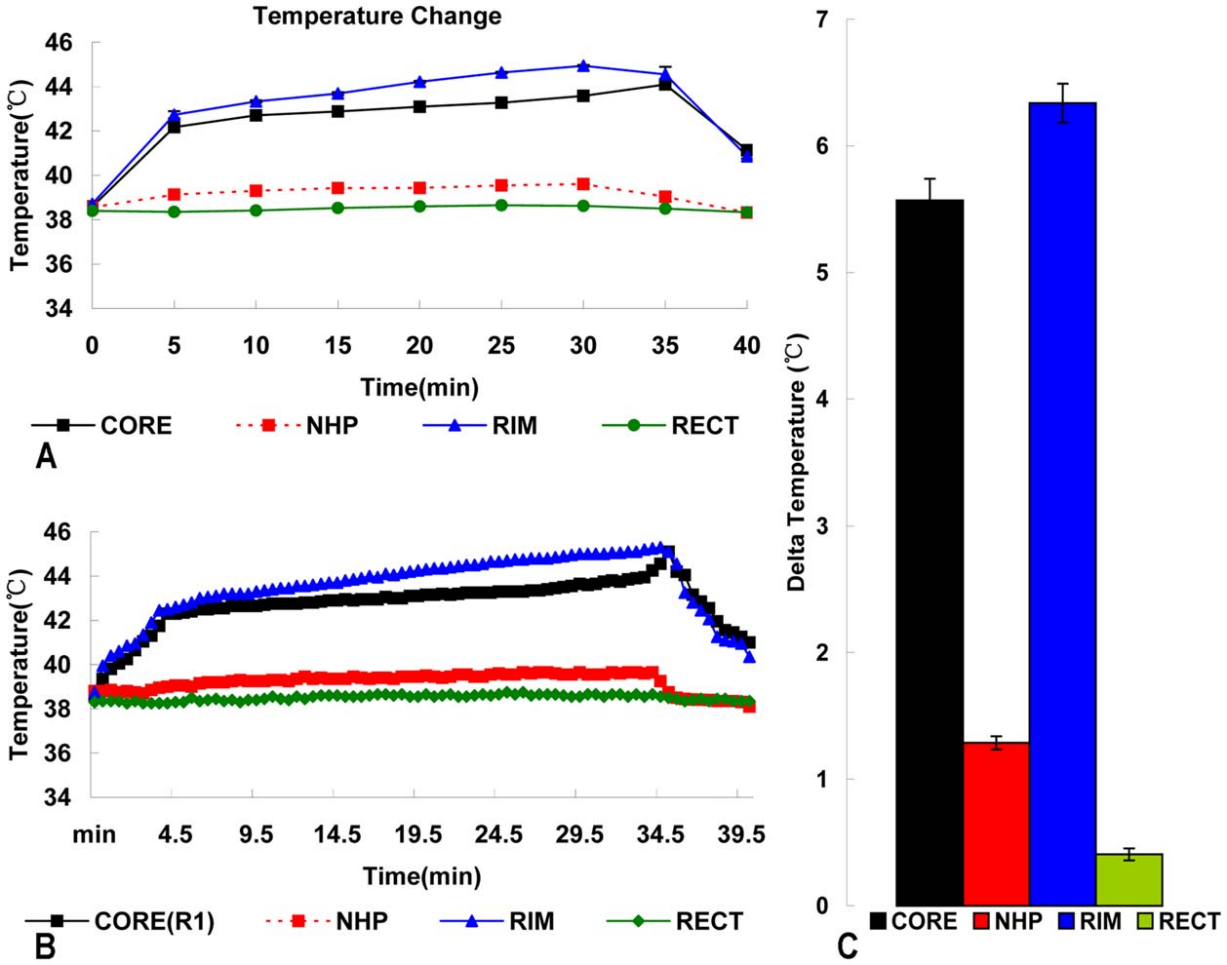
Temperature profiles during AEH treatment. Figure 4A Typical heating curve for the temperature of the tumor core, rim, normal liver tissue, and rectum of Subject 1 in Group 5, from baseline to temperature during AEH treatment. Figure 4B Typical mean heating curve for the temperature of each subject in Group 5 during AEH treatment. Intratumor and rim temperatures increase rapidly to 42.5°C within 5 min and are then maintained above 42.5°C for 30 min, while the temperature inside NHP and the rectum remain below 40°C. Figure 4C Intratumor and rim delta temperature changes show the specific heating of the tumor during treatment.

Intratumor and rim temperatures (Figs. 4A–C) rapidly increased to 42.5°C within 5 min, and were then maintained above 42.5°C for 30 min by carefully manipulating the magnetic field intensity. All nontumor heating levels were safely below 41°C during treatment. The heating rate was much greater in the center and periphery of the tumor than in normal hepatic parenchyma (NHP), and was highest at the rim of the tumor.

### Biochemical Tests

**Figure 5:**
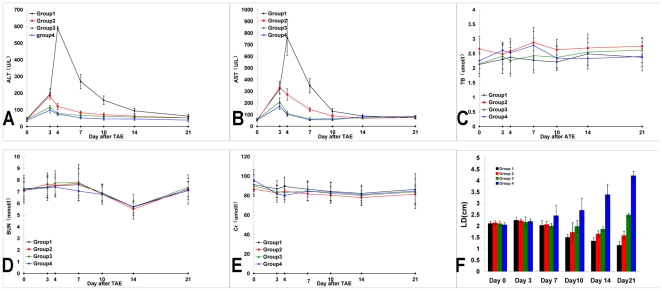
Biochemical tests and antitumor effect. Figure 5A and B The diagram shows ALT level changes in groups 1 to 4. ALT levels tended to increase significantly more in Group 1 than in the other groups 1 day after the animals were subjected to hyperthermia, but gradually decreased. AST levels changed in the same manner as did ALT levels. Figure 5C Plasma TBil levels did not change appreciably in any group. Figure 5D and E The renal function test, in which the plasma BUN and Cr values were detected, showed no statistically significant differences among the groups. Figure 5F Antitumor effect evaluated by the tumor long-axis dimensions in both the control and treatment groups. During the experimental period, the mean LD decreased significantly more in groups 1 and 2 than in the other two groups.

Liver function test results (Fig. 5A, B) showed that AST and ALT levels tended to increase transiently 1 day after intra-arterial embolization and last for several days following the infusion, regardless of which agent was administered. Plasma TBil (Fig. 5C), BUN (Fig. 5D) and Cr (Fig. 5E) levels did not change appreciably in any group. Elevated AST and ALT levels were transient and significant (P<0.05) 1 day after intra-arterial embolization and hyperthermia, but decreased to levels close to normal by 14 days after intra-arterial embolization. There were no significant differences in biochemical values among the four groups at days 14 or 21 (P>0.05).

### Treatment-associated Tumor Response

The tumor-inhibited effect as evaluated by the Response Evaluation Criteria in Solid Tumors (RECIST) methodology [18] at day 21 is shown in Table 1. A gradual decrease of the tumor's LD on CT scanning was observed during the following 3 weeks in all Group 1 rabbits treated using AEH with ATONs, and no tumor recurrence was present on follow-up CT scanning. The mean LD in Group 1 at 1 and 2 weeks post-treatment was significantly decreased compared with that in the other three groups (P<0.05, each; Fig. 5F).

**Table 1:** Tumor Response by RECIST criteria.

	Tumor Response			
	Complete Response (%)	Partial Response (%)	Stable Disease (%)	Progressive Disease (%)
Group1(n = 7)	0	7(100)	0	0
Group2(n = 8)	0	4(50)	4(50)	0
Group3(n = 7)	0	2(28.5)	2(28.5)	3(43)
Group4(n = 8)	0	0	0	8(100)

### Histopathological Findings

**Figure 6:**
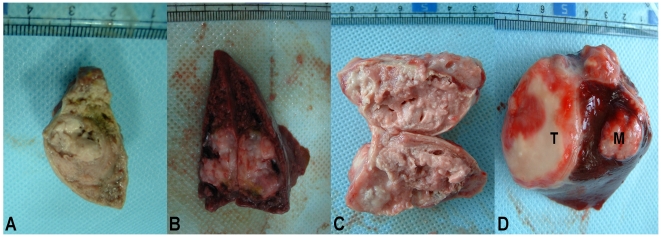
Gross pathologic specimens of response to different treatment. Figure 6A and B Gross pathologic specimens of ATON-induced necrosis in VX_2_ liver tumors treated with ATON embolization plus hyperthermia (A) and with ATON embolization alone (B). C: Lipiodol embolization resulted in significantly (P<0.05) larger tumors than those of groups 1 or 2 at day 14. D: Gross pathologic specimens of a tumor from an NS control rabbit (Group 4), showing a solitary mass (T) and intrahepatic metastasis (M) at the time of death.

**Figure 7:**
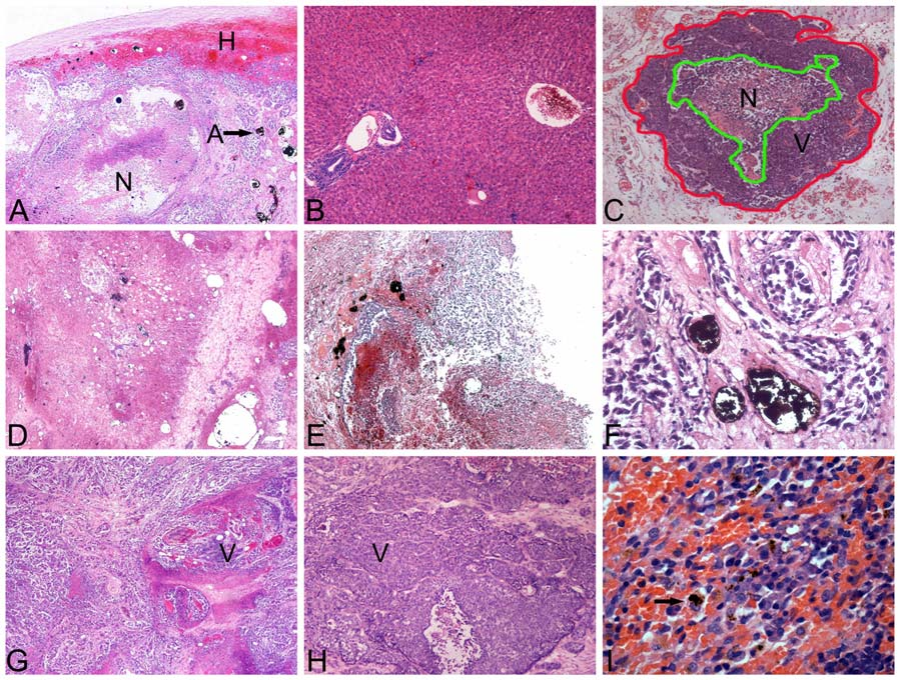
Representative histopathological (H&E) findings. Figure 7 A–I A: Original magnification: H&E, 100×. Section of a VX_2_ liver tumor treated with AEH mediated by ATONs (A) at the periphery, which was surrounded by NHP (H). A necrotic central core (N) is surrounded by an area of debris, inflammatory reaction, and fibrosis. B: Original magnification: H&E, 100×. Section through NHP 7 days after arterially embolized ATONs. The liver architecture is preserved. C: For each H&E histological image, both an outer tumor border (red line) and inner regions of necrosis (green line) were manually circumscribed to permit reference-standard histopathology-based necrosis-rate measurements. Original magnification: H&E, 25×. D: Original magnification: H&E, 100×. Microscopic image in Subject 4 from Group 2 treated with ATON embolization, showing ATONs deposited within the vascular system. Three days after embolization, an area of necrosis together with inflammatory cell infiltration and extensive fibrosis surrounding the necrotic area were evident. The area of necrosis was not as extensive as in Group 1. E: Original magnification: H&E, 100×. Pathological specimen from a rabbit in the hyperthermia group shows massive necrosis of the tumor and aggregates of ATONs that have embolized within the vascular system of the tumor. The VX_2_-implanted region in the liver has been replaced by scar tissue. Histological changes include hemorrhage, chronic inflammation, and necrosis. No viable tumor cells are evident in either the rim or center of the tumor. F: Original magnification: H&E, 200×. ATON aggregates confined within a blood vessel, surrounded by necrotic tumor cells 14 days after ATON arterial embolization. The tumor cells in the treated animal appear shrunken, and their nuclei are pyknotic. G: Original magnification: H&E, 100×. H&E-stained micrograph of a lipiodol-treated tumor at day 7 showing the presence of damaged tumor cells; note the intermixed areas of clearly viable tumor (V), indicating potential spread of the tumor beyond its circumscribed boundary. H: Original magnification: H&E, 100×. H&E-stained section of tumor from a control rabbit (Group 4) shows areas of viable tumor (V). I: Original magnification: H&E, 200×. Macrophages associated with ATON aggregates (black arrow) in the spleen 14 days after ATON arterial embolization.

Treated tumors (Figures 6A to C) were considerably smaller than untreated tumors (Fig. 6D). It was evident that the ATONs concentrated primarily around the periphery of tumors and were confined within blood vessels, while only a few penetrated the central core region, which was poorly vascularized (Fig. 7A). Massive necrosis and a thick fibrocollagenous capsule around the tumor, but no viable tumor cells, were seen in the specimen of AEH plus ATONs obtained on day 7. Adjacent liver parenchyma appeared normal (Fig. 7B). Tumors in the ATON group (Fig. 7D) had histological findings similar to those in the AEH plus ATONs group, except that completely necrotic portions were more prominent in the AEH plus ATONs group. In the iodized-oil group, partial necrosis occurred only in the central regions of tumors (Fig. 7G). In contrast, control tumors showed clusters of viable tumor cells with lighter regions of necrosis and cellular debris (Fig. 7H). No ATONs appeared in the lungs or kidneys of any specimens. Macrophages were associated with occasional ATONs aggregated in the spleen 14 days after ATON arterial embolization (Fig. 7I).

#### Quantitative analysis of microvessels

**Figure 8:**
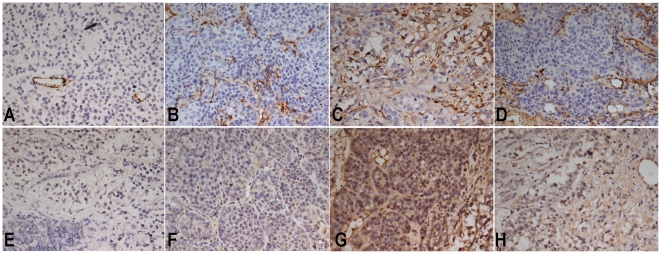
Immunohistochemical staining for CD31 and VEGF. Representative immunohistochemical staining with CD31 and VEGF monoclonal antibodies in VX_2_ liver tumors in each group. Microvessels are identified by dark brown (original magnification, 200×). A marked reduction in MVD is observed in the ATON plus hyperthermia group (A) and ATON embolization alone group (B). Abundant microvessels are evident in both the lipiodol TAE group (C) and NS control group (D). Positive VEGFs are recognizable as intensely stained in tumor cell cytoplasm (original magnification, 200×). It is evident that the abundance of VEGF-positive tumor cells is higher in the lipiodol TAE group (G) and NS control group (H) than in the ATON plus hyperthermia group (E) or ATON embolization alone group (F).

Quantitative MVD data from each group are presented in Table 2. A significant reduction in MVD was noted in Group 1, which was treated with ATONs plus hyperthermia (Figs. 8A–D).

**Table 2:** MVD and Mean Tumor Necrosis Ratios in Different Groups.

Group	Mean necrotic area ratios (%) (mean±SD)	MVD (mean±SD)
1(n = 4)	92.08±2.62^a^^,^ b ^,^ c	17.95±5.44^g^^,^ h ^,^ i
2(n = 4)	84.00±4.75^d^^,^ e	32.85±7.53^j^^,^ k
3(n = 4)	59.75±10.59^f^	76.30±19.16
4(n = 4)	44.63±10.96	63.05±11.20

Mann-Whitney U Test:

a :G1 vs. G2 *p* = 0.000;

b : G1 vs. G3 *p* = 0.000;

c : G1 vs. G4 *p* = 0.000;

d :G2 vs. G3 *p* = 0.000;

e : G2 vs. G4 *p* = 0.000;

f : G3 vs. G4 *p* = 0.000;

g :G1 vs. G2 *p* = 0.000;

h : G1 vs. G3 *p* = 0.000;

i : G1 vs. G4 *p* = 0.000;

j :G2 vs. G3 *p* = 0.000;

k : G2 vs. G4 *p* = 0.000.

#### VEGF expression

In Group 1, plasma VEGF levels were elevated at day 3 after ATON plus hyperthermia treatment, followed by a significant decrease through days 7, 10, 14, and 21 (Table 3). Group 1 also had significantly lower VEGF levels in tumors compared with Group 2 (P = 0.003; Table 4). The VEGF-positive rate was significantly lower in Group 1 than in Group 4 (P = 0.0027) or Group 3 (P<0.001). The difference between Groups 1 and 2 was not statistically significant (Table 4, Fig. 8E–H).

**Table 3:** Plasma VEGF Levels in Different Groups.

Group	Plasma VEGF Level (pg/ml)					
	Day 0	Day 3	Day 7	Day 10	Day 14	Day 21
1	64.95±19.98	95.00±40.10^b^	58.37±21.19^b^^,^ c	49.40±20.03^b^^,^ c	38.33±17.86^b^^,^ c	29.21±8.18^a^^,^ b ^,^ c
	(n = 8)	(n = 7)	(n = 7)	(n = 7)	(n = 7)	(n = 7)
2	67.01±19.29	98.08±31.47^d^	70.13±30.39^d^	54.89±20.52^d^^,^ e	45.69±15.96^d^^,^ e	44.56±13.79^d^^,^ e
	(n = 8)	(n = 8)	(n = 8)	(n = 8)	(n = 8)	(n = 8)
3	62.54±20.26	159.67±48.23^f^	176.54±27.57^f^	166.67±38.12^f^	95.48±31.19^f^	83.94±17.99^f^
	(n = 8)	(n = 7)	(n = 7)	(n = 6)	(n = 6)	(n = 5)
4	63.10±20.51	71.24±20.96	88.38±27.78	122.93±18.44	172.97±30.60	298.00±79.37
	(n = 8)	(n = 8)	(n = 8)	(n = 7)	(n = 6)	(n = 3)

Mann-Whitney U Test:

a :G1 vs. G2 *p* = 0.021;

b : G1 vs. G3 *p*<0.05;

c :G1 vs. G4 *p*<0.05;

d :G2 vs. G3 *p*<0.05;

e : G2 vs. G4 *p*<0.05;

f :G3 vs. G4 *p*<0.05.

**Table 4:** Comparisons of Tissue VEGF levels at Day 7 between groups.

Group	Tissue VEGF at Day 7(mean±SD)pg/mg protein	VEGF expression at day 7(10 sections/Tumor)			Positive Rate(%)
		−	±	+	
1(n = 4)	30.05±7.12^a^^,^ b ^,^ c	32	5	3	8/40 (20%)^g^^,^ h ^,^ i
2(n = 4)	41.22±9.25^d^^,^ e	28	5	7	12/40 (30%)^j^^,^ k
3(n = 4)	97.64±17.31^f^	28	5	7	34/40 (85%)^l^
4(n = 4)	60.85±11.16	18	16	6	22/40 (55%)

Abbreviations: −(negative); ±(weakly positive);+(positive).

Mann-Whitney U Test:

a :G1 vs. G2 *p* = 0.003;

b : G1 vs. G3 *p* = 0.000;

c : G1 vs. G4 *p* = 0.000;

d :G2 vs. G3 *p* = 0.000;

e : G2 vs. G4 *p* = 0.000;

f : G3 vs. G4 *p* = 0.000.

Chi-square test:

g :G1 vs. G2 *p* = NS;

h : G1 vs. G3 *p* = 0.000;

i : G1 vs. G4 *p* = 0.0027;

j :G2 vs. G3 *p* = 0.000;

k : G2 vs. G4 *p* = 0.0418;

l : G3 vs. G4 *p* = 0.0073.

#### Tumor necrosis

The mean tumor necrosis rates of the tumors in each group are given in Table 2. The ATON plus hyperthermia group (Group 1) showed the highest tumor necrosis rates (P<0.001).

### Survival

All rabbit deaths during the course of the study were caused by hepatic and/or respiratory failure secondary to extensive tumor burden. All of these events occurred within 57 days, reflecting the aggressive nature of this tumor model.

**Figure 9:**
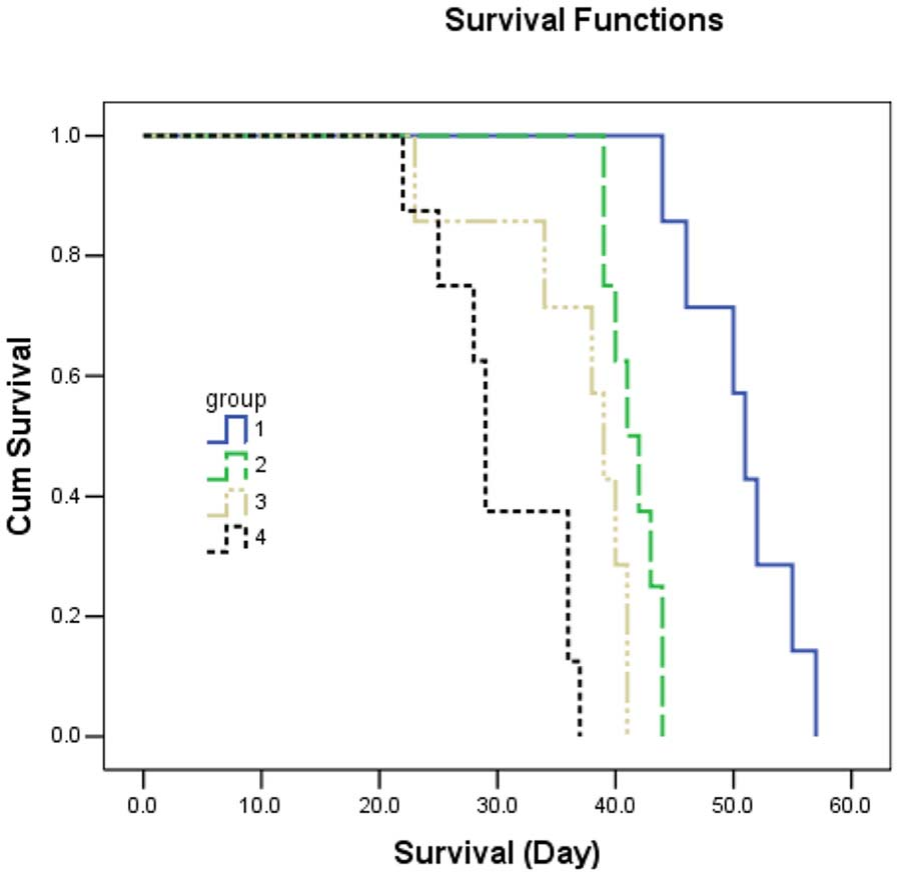
Kaplan-Meier survival analysis of liver-tumor-bearing rabbits. Graph of the Kaplan-Meier method with the log-rank test shows a significant survival benefit for the animals treated with ATON embolization alone or plus hyperthermia compared with those in Groups 3 and 4.

Even though no rabbits were completely cured, animals in the AEH plus ATON group survived significantly longer than did the controls (Kaplan-Meier method with log-rank test, P<0.001; Fig. 9).

## Discussion

Although currently available TACE may result in a modest improvement in HCC patient survival, a radiological complete response is achieved in only 35% of patients according to EASL criteria [19]. The overall prognosis of these patients remains poor [20], [21]. However, eligible patients have already benefited from available hyperthermia technologies [22], [23] including radiofrequency, which heats not only the tumor but also the surrounding healthy tissue. Another limit of radiofrequency is the cooling effect of the intact blood supply [8], which results in nonuniform heating of the target area. Thus, development of more effective therapies is urgently needed.

AEH is based on using the arterial supply of a tumor to provide a pathway for selective embolization of the tumor by using magnetic particles, followed by exposure to an alternating magnetic field to generate heating of the embolized particles by hysteresis or Néel relaxation. Briefly, Néel loss is caused by the relaxation of magnetization vector rotation of single-domain magnetic particles in an applied AC field, while hysteresis loss is heat loss caused by the magnetic properties of the armature.

As_2_O_3_ is currently being tested against various malignancies, including HCC [11]–[14], [24], [25]. However, its clinical application is limited by its narrow therapeutic window. Alternatively, ATONs deliver local treatment and potentially achieve therapeutic efficacy by improving tumor/normal tissue uptake ratios; furthermore, they avoid toxicity by reducing uptake of ATONs by nontarget organs [26]. Both the antivascular effects and direct cellular thermosensitization of As_2_O_3_ contributed to the increase in heat-induced tumor growth delay observed in these studies [24]. As_2_O_3_ reduces the blood flow *in vivo* and prolongs the dwell time of the drug within the tumor, and preferential uptake of iodized oil would be expected to increase the amount of drug directed at the tumor [27]. Thus, we believe that ATONs combined with AEH could exert a synergistic antitumor effect via two properties: chemotherapy of As_2_O_3_ and thermotherapy of magnetic Fe_2_O_3_ nanoparticles.

Our hyperthermia system consists primarily of a ferrite-core applicator with an aperture with a 350-mm vertical distance, 300-mm length, and 200-mm width (large enough to accommodate a rabbit or even larger animals). The device is operated at a frequency of 80 kHz, and the field strength can be adjusted from 5 to 10 kA/m. Our hyperthermia system can generate high temperatures. To determine the possibility of using either AMF or lipiodol in heating, a preliminary experiment was performed with another group of animals using saline or lipiodol. A FISO probe was introduced into the tumor, showing that the AMF was activated; we had to exclude the possibility that the magnetic field itself was contributing to any change in temperature or tumor growth (data not shown).

Over the last decade, several studies [9], [28]–[30] have investigated the potential of various magnetic nanoparticles, including maUgUnetite cationic liposomes, γ-Fe_2_O_3_, and Fe_3_O_4_ in AEH. Our study is the first report on the therapeutic effects of ATONs in an animal HCC tumor model. In a previous study, we evaluated the different heating abilities of γ-Fe_2_O_3_ nanoparticles and ATONs. The magnetic properties of the nanoparticles were shown in a hysteresis loop (Fig. S1), which indicated that γ-Fe_2_O_3_ had a higher specific absorption rate (21.4×10^3^ W/kg Fe) than ATONs (18.2×10^3^ W/kg Fe) under an alternating current magnetic field of 10 kA/m and 80 kHz. However, both nanoparticles achieved therapeutic temperatures in the *in vivo* experiment.

Our self-prepared ATONs combined with hyperthermia had a significant therapeutic effect on VX_2_ liver cancers. Tumor growth was delayed, angiogenesis was inhibited, survival time was prolonged, and side effects were endurable. Our promising results are consistent with and extend a previous study [31], which showed that a combination of nanosized As_2_O_3_/Fe_2_O_3_ complexes has a substantially greater inhibitory effect than either agent alone. The findings from our present study may be explained by synergy between ATON embolization and hyperthermia. ATON embolization is able to block the flow of blood to a tumor, reduce tumor perfusion, and decrease heat dissipation [24], [25], which further enhances the hyperthermic therapeutic effect, and inhibit angiogenesis [17], [32]. The antiangiogenic effect of ATON embolization plus hyperthermia was demonstrated by quantitative MVD analysis and ELISA analysis of decreased VEGF expression in tumors and plasma.

Our results indicate that AEH using ATONs can specifically heat tumor tissue without damaging adjacent healthy tissue. It has been demonstrated that tissue iron concentrations are linearly related to heating rates [9], [27]. In our study, the higher concentration of ATON embolization in the highly vascularized tumor rim (Fig. 7A) compared with that in the poorly vascularized tumor core led to higher temperatures in the tumor rim than in the tumor core or normal tissue (Fig. 4). This targeted hyperthermia was due to two factors. First, infusion of the embolized agent into the hepatic arterial system resulted in the deposition of ATONs throughout the tumor, excluding regions of necrosis, thereby targeting the tumor and largely sparing NHP. Second, the half-life of lipiodol is greater in a tumor than in NHP. The significantly elevated temperature at the core of tumors was not surprising. The blood vessels of tumors are dilated, tortuous, and without complete basement membranes. Furthermore, venous drainage of tumors is often chaotic and incomplete [8]. These factors are likely to result in better heat conduction and poorer cooling mechanisms due to poorer blood perfusion (as a result of the absence of an ordered, high-flow, portal venous system) within the necrotic regions of tumors. Poorer blood flow through the center of tumors may favor the flow of heat into the tumor core from the hotter rim. Previous results [33] support this assertion.

The distribution and clearance of ATONs are important issues for the potential clinical application of ATONs. We injected ATONs into the hepatic artery; most ATONs went to targeted liver tumors. ATONs deposited in either normal liver or tumor tissue were still clearly visible on CT images or pathology slices several weeks after ATON injection. The long deposition time of ATONs in NHP may lead to unexpectedly high temperatures in healthy tissue during treatment. However, clearance of ATONs was much faster from tumors than from NHP. Therefore, we implemented hyperthermia 3 days after TAE. This reduced the potential threat of liver dysfunction in rabbits treated with TAE plus hyperthermia. However, in a clinical setting, superselective catheterization of the tumor-feeding artery could be performed before arterial embolization and hyperthermia, which would spare as much normal liver tissue as possible and reduce ATON deposition, thus preventing this potential complication. On the other hand, sparse distribution of ATONs in NHP cannot be avoided, but the large portal blood flow would cool these point sources of heat and thus prevent thermal damage to NHP. Our results demonstrate that the temperature of NHP remained at safe levels (below 41°C) during treatment. Furthermore, our results are consistent with those of Moroz et al. [9], who showed that the lack of a raised temperature in the rectum indicates that no significant body core heating has occurred. This indicates that either no ATONs passed through the hepatic circulation into the systemic circulation or no significant nonhepatic tissue heating occurred.

Histological analysis of lung and kidney tissues failed to show evidence that ATONs passed through the liver and lodged within the lungs or kidneys. The finding of ATON aggregates within macrophages in the liver and spleen suggests that ATONs may be eliminated from the liver and spleen over time. Examination of splenic tissue (day 14) revealed some macrophages in association with ATONs, while splenic tissue at days 1, 3, and 10 failed to show them, presumably indicating that ATONs were not removed by macrophages before day 14 after TAE. This may be an advantage, because this time interval allows for a sufficient number of repeat heat treatments which can enhance hyperthermic effect. We believe that it represents an important prerequisite for the clinical application of this technique in liver cancer.

AEH has not yet been tested in humans, and the clinically relevant dosage remains largely unknown. The net γ-Fe_2_O_3_ dose (21.2 mg/kg body weight) applied in our regional procedure was similar to those of previous preclinical hyperthermia studies, which showed effective inhibition of tumor growth by hepatic arterial administration of nanoparticles at doses of 25–100 mg in each rabbit with hepatic orthotopic VX_2_ carcinoma [8], [9], [27]. On the other hand, the net As_2_O_3_ dose (2.8 mg/kg body weight) applied in our study was clearly within the dose range of previous studies, which used As_2_O_3_ to treat experimental solid tumors after single or multiple doses of 1–10 mg/kg [16], [24], [25], [34]–[40]. However, such doses are considerably higher than the daily dose of 0.16–0.24 mg/kg given for several weeks in a recent phase II clinical trial, which was not active against advanced HCC [41]. Our doses were chosen on the assumption that a human liver weighs 15 times that of a rabbit liver (1500 g compared with 100 g) [8], [9]. Thus, doses of 24 mg/kg ATONs (0.3 mL suspension/kg, total 0.9–1.2 mL/rabbit) in a rabbit would be equivalent to a total of 13.5–18 mL of ATON suspension in a human subject. These volumes are comparable with those currently used in TACE. In TACE, up to 20 mL of lipiodol containing a chemotherapeutic agent may be used to embolize liver tumors via the hepatic arterial system. Although the present preliminary study has shown promising results, the optimal ATON dose that is appropriate for human investigations is unclear at this time, and further studies are needed.

Our study has several limitations. First, in terms of the diameter and dosage of ATONs, the power of the magnetic induction field, and the duration of heating, our *in vivo* experiments were conducted under only one set of conditions. Second, the therapeutic effect of ATONs was not compared with that of other chemotherapy drugs or other magnetic nanoparticles. Third, we did not investigate the circulation of ATONs in the blood. However, on the basis of excellent tumor targeting, local arterial embolization could guarantee an increase in the local tumor concentration of ATONs while reducing the amount of circulating ATONs. Despite these limitations, our preliminary study demonstrated that AEH mediated by ATONs has a sound theoretical basis, and it produced some encouraging results that could ultimately be integrated into clinical practice.

### Conclusions

AEH with ATONs inhibited tumor growth in most treated animals with temporal hepatic dysfunction, and significantly improved survival in the intrahepatic VX_2_ tumors in our study. The therapeutic effect of AEH may be further enhanced when it is combined with ATONs, the antitumor effects of which are partly due to inhibition of tumor angiogenesis but also correlated with altered VEGF expression in the tumor and plasma. Further studies aimed at elucidating the optimal dose and mechanism in animals, and ultimately clinical trials, are required.

## Materials and Methods

### Animal Models and Groups

All animal procedures were performed in accordance with the approval and guidelines of the Institutional Animal Care and Use Committee (IACUC) of the Medical School of Southeast University (approval ID: SYXK-2007.2121).

**Figure 10:**
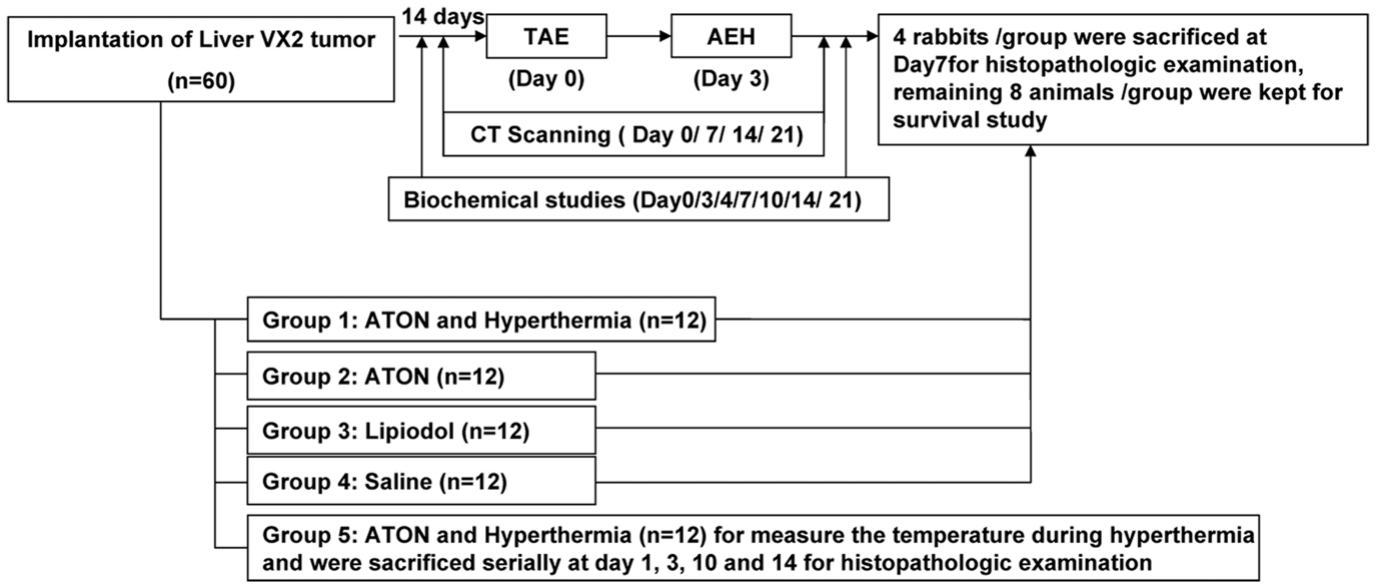
Flow chart of the experiment. Flow chart of the experiment and diagram showing experimental groups according to the embolization agents that were used for intra-arterial delivery.

Sixty male New Zealand white rabbits (provided by Southeast University, Nanjing, China) were randomly assigned to five groups (*n* = 12 each; Fig. 10). An orthotopic model of hepatocarcinoma was developed using VX_2_ carcinoma as described previously [42]. The animals from each group received a hepatic arterial infusion of arsenic trioxide (As_2_O_3_) nanoparticles (ATONs) (Groups 1, 2, and 5), lipiodol (Group 3), or saline (Group 4). In Groups 1 and 5, hyperthermia was induced on day 3 after transcatheter arterial embolization (TAE). Four rabbits from Groups 1 to 4 were sacrificed on day 7 for histopathological examination after TAE, whereas the remaining eight animals in each group were kept for the survival study. Animals in Group 5 were used for dynamic measurement of temperatures during the hyperthermia procedure, and were serially sacrificed at 1, 3, 10, and 14 days after TAE (three rabbits each day) to verify the distribution of ATONs.

### Treatment Procedure, Temperature, and Tumor Response Measurement

TAE was performed (H.Y., G.Y.Z.,) at 14 days after tumor implantation using an angiography system (Innova 3100, GE Medical Systems, Milwaukee, WI, USA) according to a method described previously [43]. Briefly, using an aseptic technique, the right femoral artery of an anesthetized rabbit (1% sodium pentobarbital at 4 mL/kg intravenous infusion, Shanghai Chemical Reagent Co., Shanghai, China) was accessed via surgical cutdown and a 4F vascular sheath was placed (Terumo, Tokyo, Japan). Selective catheterization of the proper hepatic artery feeding the VX_2_ carcinoma was performed using a 3F microcatheter (SP, Terumo, Tokyo, Japan), which was coaxially inserted through a 4F Cobra catheter (Cook Inc., Bloomington, IN, USA) with fluoroscopic guidance. Hepatic angiography was obtained with hand injection of 3 mL of contrast medium (Omnipaque 300; Ansheng Pharmaceutical Co., Shanghai, China) that was injected at a rate of approximately 0.5 mL/s.

As_2_O_3_ nanoparticles (ATONs; approximately 40 nm in diameter; 88.50% (wt/wt) γ-Fe_2_O_3_ Hcore coated withH 11.50% (wt/wt) As_2_O_3_) were made in our laboratory according to a method described previously [31], [44]. A thin layer of ATONs was spread and exposed to ultraviolet light for sterilization. The ATONs were then magnetically separated and quantitatively dissolved in lipiodol (Laboratoire Guerbat, France; 80 mg ATONs/1 mL lipiodol) via ultrasonography.

After the desired ATON suspension dose (24 mg ATONs, including net γ-Fe_2_O_3_ 21.2 mg plus net As_2_O_3_ 2.8 mg per kg body weight) had been carefully infused through the 3F microcatheter into the tumor-feeding artery, the femoral artery was ligated and the wound was closed. Selective catheterization of the tumor-feeding artery under a DSA monitor guaranteed tumor target embolization and spared arterial flow through the hepatic artery into the liver.

**Figure 11:**
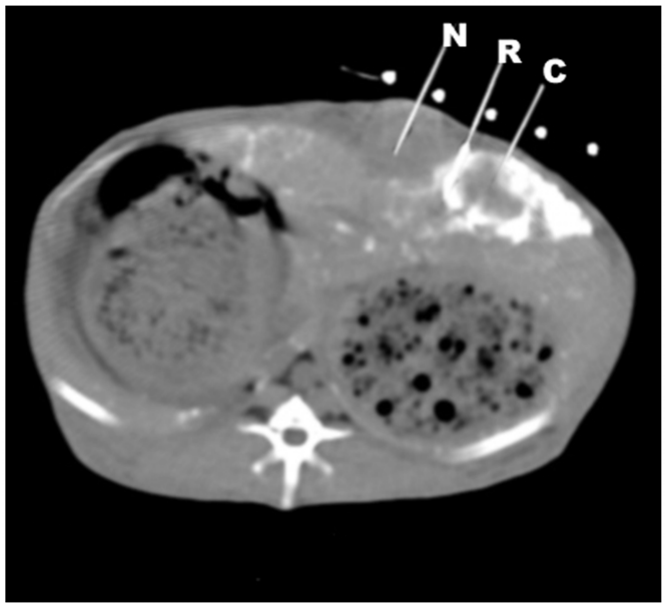
CT-guided percutaneous puncture to monitor temperature changes. Under CT-guided percutaneous puncture, implantation of three 18G puncture needles and replacement of FISO probes using the coaxial method were performed. The probes were located in the core (C), rim (R), and NHP (N) 1 cm away from the rim to monitor temperature changes in real-time, and the animals were subsequently transferred to AMF for hyperthermia.

**Figure 12:**
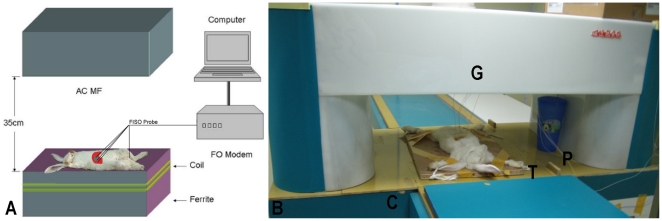
Arterial embolization hyperthermia therapy system. Figure 12A Scheme of AEH therapy system and method of temperature measurement *in vivo*. Four FISO probe fibers that were attached to the inside wall of a plastic tube connected to a computer were used to detect temperature changes in the tumor core, tumor rim, and 1 cm from the tumor and rectum. Figure 12B Apparatus of new arterial embolization hyperthermia therapy system for large animals or humans. The system comprises a generator (G), induction coil (C), treatment table (T), and FISO probe (P).

Three days after TAE, using the general anesthesia protocol and aseptic technique described above, a puncture needle (18G, Terumo, Tokyo, Japan) was percutaneously advanced into the center or the rim of the tumor, the normal liver 1 cm from the tumor rim, and the rectum, under CT guidance (Fig. 11). The needle stylet was then replaced with a fiberoptic temperature probe (FISO Inc., Quebec, QC, CA). This CT-guided percutaneous puncture and temperature measurement technique minimized potential heat loss to the external environment. Animals were subjected to the AEH therapy system (Figs. 12A, B) described previously (frequency, 80 kHz; field strength, 5–10 kA/m) [28], [29]. Each probe tip temperature, wattage, field intensity, and frequency was recorded during the procedure. After the tumor core temperature had reached the intended temperature (42.5°C), it was maintained at 42–45°C for a period of 30 min by manually adjusting the magnetic field strength. The temperature of the liver itself was maintained at approximately 37.8°C.

Plain CT scanning was performed to evaluate the ATON distribution and each tumor's longest dimension (GE HiSpeed CT/i Scanner, GE Medical Systems, Milwaukee, WI, USA) at days 0, 3, 4, 7, 10, 14, and 21 with parameters of 120 kV, 200 mA, FOV 16 cm, slice thickness 3 mm, pitch 1.5 mm, reconstruction 2 mm, and matrix 512.

Response Evaluation Criteria in Solid Tumors (RECIST) criteria were used to determine objective responses to treatment [18], [45], [46]. A complete response (CR) was defined as the disappearance of all lesions with no new lesion development; a partial response (PR) as a decrease of 30% or more in the longest dimension (LD) or the sum of the longest dimensions of all measured target lesions; stable disease (SD) as any change in tumor size that did not satisfy PR or PD criteria; and progressive disease (PD) as an increase of >20% in the longest dimension or the sum of the longest dimensions of all measured target lesions or any new lesion. All CT images were blindly analyzed by two radiologists (Q.L. and H.Z.N., who had 15 and 12 years of experience, respectively) by consensus.

### Biochemical Studies and Vascular Endothelial Growth Factor Assay

Peripheral blood samples (2.0 mL) were collected for biochemical examination at days 0, 3, 4, 7, 10, 14, and 21 after treatment. Plasma aspartate aminotransferase (AST), alanine aminotransferase (ALT), blood urea nitrogen (BUN), Hserum creatinineH (Cr), and total bilirubin (TBil) levels were measured using a biochemical autoanalyzer (Model LX 20; Beckman, CA, USA).

The tumor tissues were prepared as described previously [47]. Vascular endothelial growth factor (VEGF) levels of plasma and tumor tissue were measured using an ELISA assay (Rapid Bio, CA, USA) according to the manufacturer's instructions. Each sample was measured in triplicate.

### Histopathological and Immunohistochemical Examinations

The animals were sacrificed by intravenous injection of an overdose of sodium pentobarbital at the defined endpoint. The intact liver, lungs, kidneys, spleen, and any metastases were removed and fixed in 10% buffered formalin, and were then carefully sectioned in 4-µm-thicknesses in the axial plane to correspond with the plane of the CT scan. All paraffin-embedded, hematoxylin and eosin (H&E)-stained sections were examined microscopically.

To assess VEGF expression, 10 random non-necrotic areas (×200) from each specimen were evaluated [48] using a VEGF monoclonal antibody (Santa Cruz Biotechnology Inc., CA, USA). Experiments were performed according to the supplier's instructions. VEGF expression was semiquantitatively evaluated at three levels: positive staining in less than 10% was regarded as negative (−), positive staining in 10–50% as weakly positive (±), and positive staining in 50% or more as positive (+) [49]. For determination of MVD, paraffin-embedded sections were stained with an anti-CD31 rabbit monoclonal antibody (DAKO Corp., Carpinteria, CA, USA) following a standard SABC procedure, according to a method described previously [50]. Briefly, five fields of “vascular hot spots” with a 200-fold magnification in each tumor section obtained at 7 days after TAE were examined, and the mean MVD value was recorded in a blinded fashion. The percentage of the necrotic area in the entire tumor area was calculated from H&E sections according to a method described previously [30].

Histopathological slides were examined with a Scope.A1 microscope (ZEISS, Germany) equipped with an Axiocam MR5 digital camera and Image-Pro Plus version 6.0 (Media Cybernetics, Silver Spring, MD). An experienced pathologist (Z.Y.W) who was blinded to the experiment details evaluated all specimens.

### Statistical analyses

Statistical evaluation was performed using SPSS software (ver. 13.0; SPSS Inc., Chicago, IL, USA). Numerical data were expressed as means±SD. A P value<0.05 was considered to indicate statistical significance. The Kruskal-Wallis and Mann-Whitney U tests were performed among groups. Survival rates were assessed using the Kaplan-Meier method.

## Supporting Information

Figure S1 **The magnetic properties of γ-Fe_2_O_3_ nanoparticles and ATONs.** The magnetic properties of γ-Fe_2_O_3_ nanoparticles and ATONs were shown in a hysteresis loop according to a method described previously [28], [29], indicating a higher specific absorption rate of γ-Fe_2_O_3_ nanoparticles than of ATONs under an alternating current magnetic field of 10 kA/m and 80 kHz.
(TIF)
