# Supplementary figures and images for: Arterial Embolization Hyperthermia Using As2O3 Nanoparticles in VX2 Carcinoma–Induced Liver Tumors
Source: PLoS One. 2011 Mar 23;6(3):e17926. doi: 10.1371/journal.pone.0017926 (PMC3063167; doi:10.1371/journal.pone.0017926)

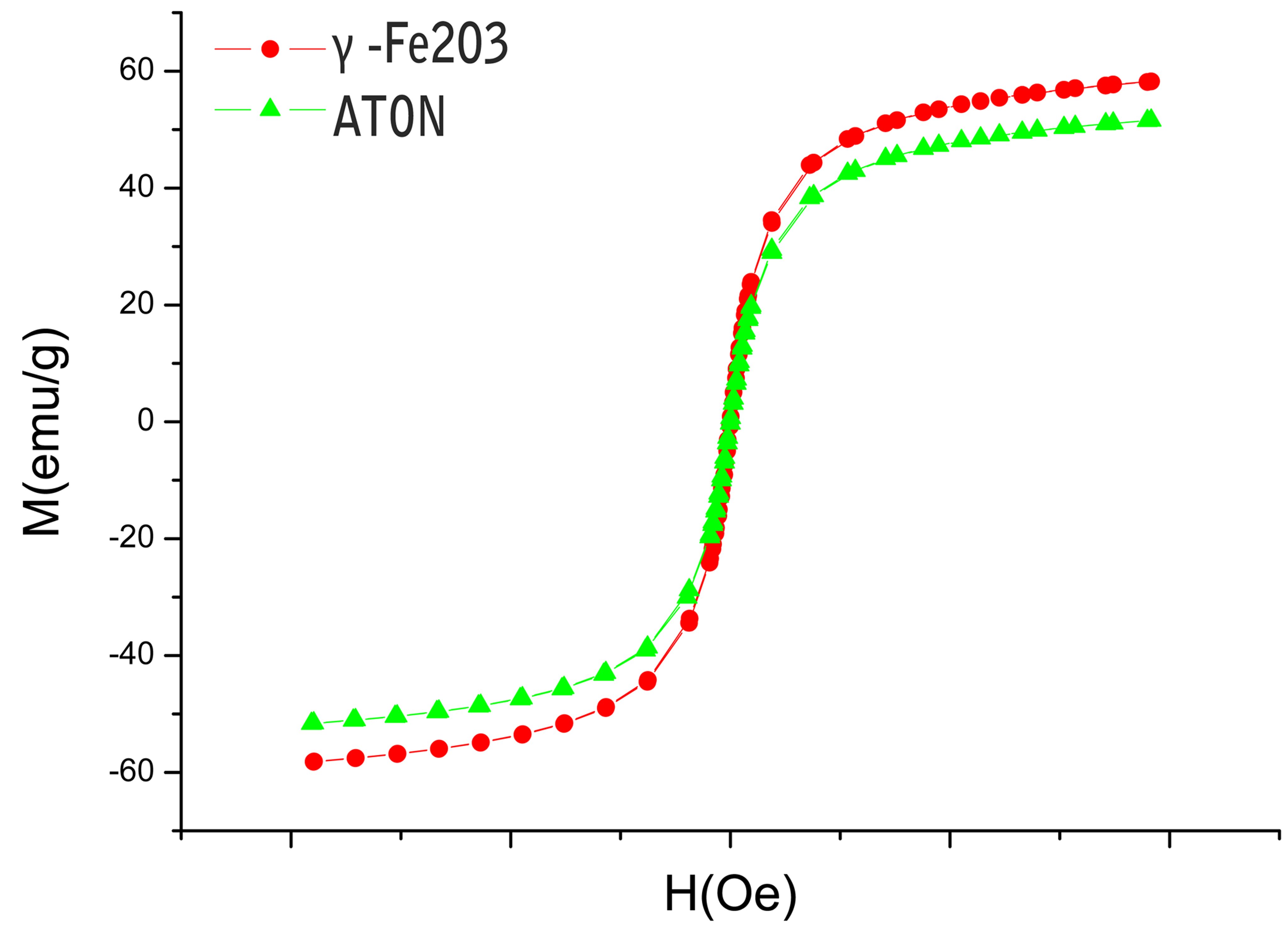

Supplement: Figure S1 — The magnetic properties of γ-Fe2O3 nanoparticles and ATONs. The magnetic properties of γ-Fe2O3 nanoparticles and ATONs were shown in a hysteresis loop according to a method described previously [28], [29], indicating a higher specific absorption rate of γ-Fe2O3 nanoparticles than of ATONs under an alternating current magnetic field of 10 kA/m and 80 kHz. (TIF) [file pone.0017926.s001.tif]
